# Supplementary material for: COVID-19 incidence and outcome by affluence/deprivation across three pandemic waves in Ireland: A retrospective cohort study using routinely collected data
Source: PLoS One. 2023 Jul 21;18(7):e0287636. doi: 10.1371/journal.pone.0287636 (PMC10361474; doi:10.1371/journal.pone.0287636)
Supplement: Attachment — Submitted filename: PLOS ONE RESPONSE TO REVIEWERS 06062023.docx [file pone.0287636.s002.docx]

|  | National Health Intelligence Unit  Health Service Executive Research and Development  4^th^ Floor Jervis House  Jervis Street  Dublin D01 W596 |
| --- | --- |

**Response to reviewers**

**PLOS One**

**Ref: [PONE-D-22-14948R1]**

**6^th^ June 2023**

Dear colleague

Very many thanks for your recent editorial advice. The only point outstanding was number 6, which I have included in full below:

**6. Review Comments to the Author**

Reviewer #1: Thank you for the revision in response to my comments. I found all comments have been fully addressed. I have no further questions.

Reviewer #2: I was satisfied with all of the responses to my comments except one.

"Comment: I like Figure 3 very much and it seems to suggest important interactions between age and deprivation and perhaps age, deprivation, and wave. Were interactions considered in the model? It would seem that some should be given the exploratory data analysis.

Response: Interactions were considered in the model, particularly interaction between deprivation and age, and that is controlled for in the model."

The authors state that they have considered interactions and that an interaction is controlled for in the model. Presumably that means that the model with the interaction is presented in the manuscript. The methods and results about the multivariable analysis do not mention interactions. Further, if the model included an interaction between deprivation and age then the aORs would be presented separately for the different combinations of age group and deprivation. That is, there should be an OR for age 40-49 HP1&HP2, age 40-40 HP4&HP5, etc. Having age group and deprivation entered as just main effects and not as an interaction means that there was no interaction in the model. As the authors state that there were interactions this statement and there methods and results presented are not in agreement and need further clarification.

I have had the opportunity to discuss this more closely with my colleagues and to seek additional epidemiological opinion on the issue of interaction. Because of the potential for interaction between age and deprivation, we agreed that we should include a specific interaction term including these two variable, and to determine the extent to which it may influence the overall model.

While we did observe some variable interaction, this only achieved significance for the hospital admission outcome. The overall impact on the three outcomes was substantively unchanged. In other words, age still had the greatest impact on hospital admission and mortality, and the second greatest impact on ICU admission after comorbidity, even after the model was corrected for the age*deprivation interaction.

I have amended Table 2 to include the interaction terms and have also amended the text (please see *Track changes* version) to draw readers’ attention to the interaction.

I hope that this helps. Very many thanks once again for the opportunity to explore this data in a more robust manner, as I believe it helps to reinforce the findings which we have observed.

Very best wishes and many thanks as ever for your ongoing interest and support.

**Dr. Declan McKeown MB MPH MFPHMI**

**Email**: [declan.mckeown@hse.ie](mailto:declan.mckeown@hse.ie); **Telephone**: +353872989742

Specialist Public Health Physician and Medical Epidemiologist

*On behalf of all authors*
